# Multilocular Thymic Cyst with High F^18^ Fluorodeoxyglucose Uptake and Rheumatoid Arthritis: A Case Report

**DOI:** 10.3390/jcm14020620

**Published:** 2025-01-18

**Authors:** Francesco Ferrante, Camilla Poggi, Anastasia Centofanti, Rita Vaz Sousa, Valerio Sebastianelli, Antonio Pio Evangelista, Francesco Mattoccia, Beatrice Zacchini, Tiziano De Giacomo, Marco Anile, Federico Venuta, Massimiliano Bassi

**Affiliations:** Division of Thoracic Surgery, Policlinico Umberto I, Sapienza University of Rome, 00165 Rome, Italyvalerio.sebastianelli@uniroma1.it (V.S.);

**Keywords:** multilocular thymic cyst, rheumatoid arthritis, thymus, immunoregulation

## Abstract

**Background:** multilocular thymic cysts are uncommon acquired cysts in the anterior mediastinum caused by incomplete thymic involution. They may be associated with autoimmune diseases, such as rheumatoid arthritis and systemic sclerosis. **Methods:** a 61-year-old man with a history of rheumatoid arthritis for 8 years was referred to our unit because of a multiloculated mass in the anterior mediastinum with a high F^18^ fluorodeoxyglucose uptake at PET-CT scan. Histology showed a multilocular thymic cyst with lymphoid tissue, organized in germinal centers and internodal areas. **Results:** rheumatoid arthritis-related symptoms progressively disappeared after the excision of the mass. **Conclusions:** to our knowledge, this is the first report in the English language of rheumatoid arthritis symptoms improvement after multilocular thymic cyst surgical removal.

## 1. Introduction

Multilocular thymic cysts (MTC) are uncommon acquired cysts located in the anterior mediastinum caused by incomplete thymic involution [1]. Thymic cysts account for 1–2% of anterior mediastinal cysts and are divided into congenital and acquired. The congenital ones are generally unilocular and the adjacent thymic tissue is atrophic. Inflammation is generally lacking [2]. Rarely, the congenital cyst can also result from abnormalities of branchial cleft structures [3].

The MTC probably originates from the cystic degeneration of Hassall corpuscles due to various acquired inflammatory processes [2,4,5], malignancies, and immunological impairments [6]. Suster and Rosai [4] observed a series of 18 MTCs of anterior mediastinum known not to be associated with Hodgkin’s disease or seminoma. These authors were among the first to shed light on the histological and pathogenetic origin of these cysts. They described the histological findings (“multiple cystic cavities partially lined by squamous, columnar, or cuboidal epithelium; severe acute and chronic inflammation, necrosis, hemorrhage, and cholesterol granuloma formation; reactive lymphoid hyperplasia with prominent germinal centers”) and, therefore, formulated the hypothesis that cyst formation was induced by various acquired inflammatory triggers. The tissue origin was attributable to structures derived from the epithelium of the medullary ducts of which Hassall corpuscles (HC) are a part. This mechanism of formation is shared with other cysts of the head–neck district.

Berthelot et al. [7] noticed that Filaggrin and the Fc portion of the immunoglobulin, which are two major self-antigens in rheumatoid arthritis (RA), colocalize in HC cytoplasm as if these structures were involved in immune tolerance disruption toward these antigens or as if the cells that are composed of HC could play a role in antigen-presenting before degeneration.

RA is a systemic inflammatory disease with predominant manifestations at the joint level and several features of autoimmunity; cardiovascular and pulmonary symptoms are also described. Although the pathogenesis is unknown, lymphocyte activation, immune tolerance, and interaction with antigen-presenting cells are probably involved [8].

The MTCs are reported to be associated with autoimmune diseases, such as RA and systemic sclerosis (SS), but their correlation with rheumatological symptoms remains unclear [1].

We hereby present the case of a rare metabolically active MTC in an RA patient showing a progressive decrease in RA symptoms after surgical excision.

## 2. Case Presentation

A 61-year-old man with an 8-year-long history of RA was referred to our unit with a Computed Tomography (CT) scan performed for pneumonia follow-up. The CT scan showed a multilocular mass in the anterior mediastinum, measuring 40 × 16 mm.

The patient had a history of poorly controlled rheumatologic symptoms despite several therapeutic regimens including corticosteroids, hydroxychloroquine, and methotrexate. The patient’s symptoms started about ten years prior, with morning stiffness, and recurrent and migrating arthralgia of wrists, ankles, and shoulders, treated with nonsteroidal anti-inflammatory drug (NSAID). Two years later the patient was referred to a rheumatologist for exacerbation of symptoms; a positivity of rheumatoid factor and a high titer (299 U/mL) of anti-citrullinated protein (anti-CCP) antibodies were found. Articular ultrasound showed exudative synovitis in the right wrist, in the first metatarsophalangeal joint with synovial hypertrophy bilaterally, and hyperechogenic spots in Achilles’ tendons bilaterally. No erosions were reported on radiographic imaging. Methotrexate, Leflunomide, and Adalimumab were used at different moments, with partial resolution of symptoms, but poorly tolerated by the patient in the long term. Seven months before reaching our attention, Methotrexate and Adalimumab were discontinued due to the occurrence of pneumonia. At the presentation, the current therapy was 16 mg of Methylprednisolone per day, the disease’s burden was mild, and the inflammatory indexes were normal. No smoking or alcoholic habit was reported. His medical history was marked for osteoporosis with a lumbar spine T-score of −2.9 in treatment with Denosumab and vitamin D supplementation.

Laboratory examination showed 12.65 × 10^9^/L white blood cells (neutrophil 86.6%, lymphocytes 9.5%), C-reactive protein 5300 μg/L, lactate dehydrogenase (LDH) 190 U/L, blood glucose 92 mg/dL, normal thyroid hormones levels, parathormone 38.5 pg/mL, vitamin D 33.25 ng/mL, and negative serology for HBV and HCV. A cardiologic evaluation was performed and showed no electrocardiographic and echocardiographic abnormalities. Respiratory function tests were normal.

**Figure 1:**
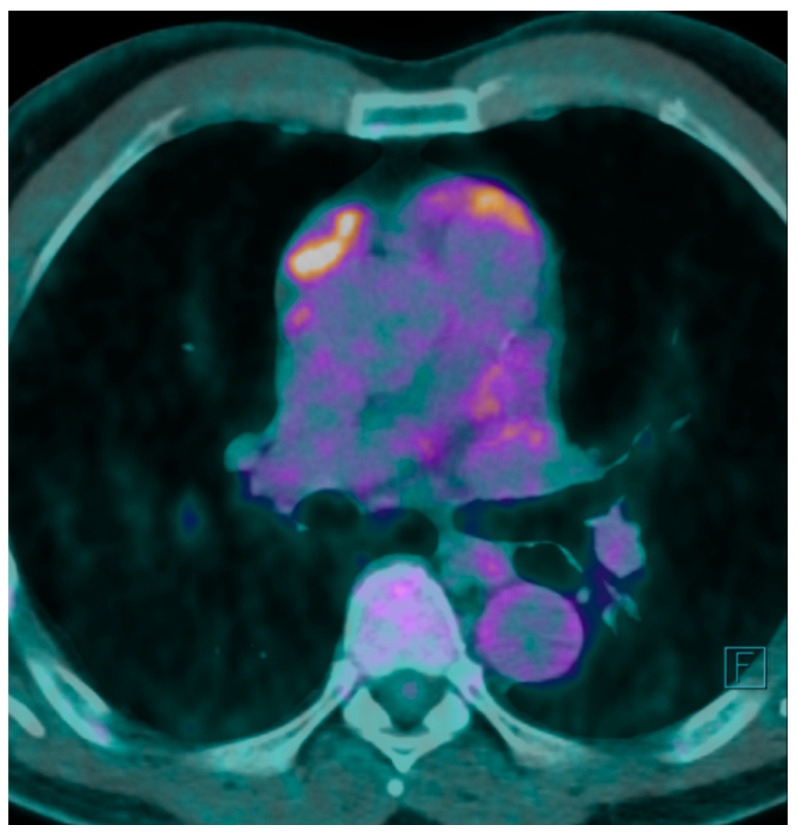
PET-CT shows uptake of F^18^ FDG in correspondence with anterior mediastinal mass. Brighter colors such as white, yellow and orange denote a higher F^18^ FDG uptake.

A F^18^ fluorodeoxyglucose-positron emission tomography (F^18^ FDG-PET) scan was performed showing a maximum F^18^ FDG Standard Uptake Value (SUV) of 7.7 at the level of the mass. The FDG uptake appeared to be heterogeneous for the presence of photopenic areas mainly due to the cystic aspect of the mass (Figure 1).

Considering the radiological suspicion of a thymic neoplasm, the patient was scheduled for mediastinal mass excision. No further investigations, such as tumor markers assay, were deemed necessary. The surgery was performed with a right uniportal video-assisted thoracoscopic approach. Intraoperatively, the mass appeared to be tightly adherent to mediastinal fat tissue with a multicystic brownish external wall. The surgical resection was complete, the postoperative course was uneventful, and the patient was discharged on the third postoperative day. At 2-week follow-up, blood tests showed normal inflammation indexes and normal leukocyte formula.

**Figure 2:**
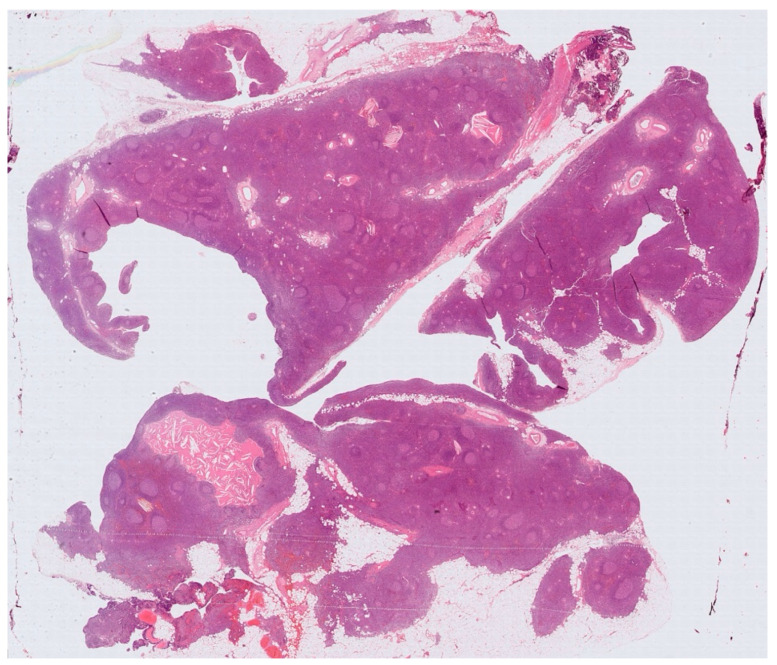
A multilocular cyst with a wall composed of lymphoid tissue organized in follicular structures.

**Figure 3:**
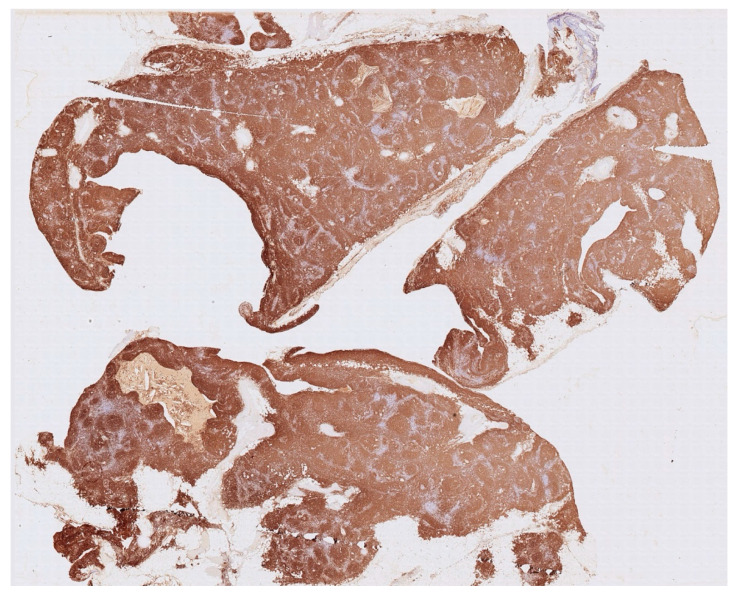
Immunohistochemistry for CD20 shows the nodular architecture of the lymphoid tissue with T cell (CD20-negative) in the internodular zone (light areas between brown zones).

**Figure 4:**
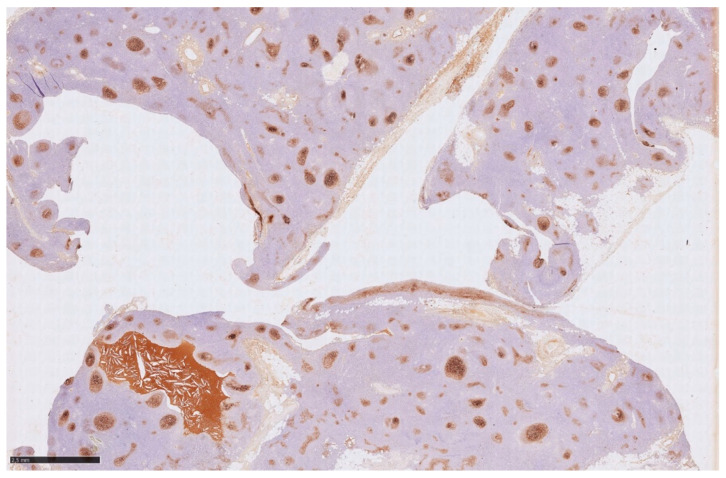
Immunohistochemistry for CD23 highlights that the CD20+ nodules are follicles (brown areas), with germinal centers, revealing the dendritic cells meshwork.

Histology showed an MTC lined by cubic epithelium and rich in lymphoid tissue, organized in germinal centers (CD10+, BCL6+, BCL2−, CD21+, CD23+), and a mantel zone (CD79A+, BCL2+), the interfollicular region represented by T-cell (CD3+), and occasional Hassall’s Corpuscles (Figure 2, Figure 3 and Figure 4).

Differential diagnosis cannot fail to consider thymic hyperplasia with lymphoid epithelial sialadenitis (LESA)-like features (LESA-like TH) [9,10]. This pathological entity presents a strong association with lymphomas and non-myasthenic autoimmune diseases, as stated by the authors. LESA-like TH is described as a tumorous proliferation, unlike MTC, and it is characterized by lymphoepithelial lesions, Hassall corpuscles with cystic changes, cortical atrophy, and lymphofollicular hyperplasia. In this disease, the appearance is compared to a lymph node with various cystic components, while in MTC, the cystic aspect is predominant in macroscopic and microscopic appearance; LESA-like TH might be diagnosed as a cystic lesion of the thymus because of a massive cystic prevalence appearance. It is undeniable that there might be some diagnostic overlap, especially because of the association that both diseases have with lymphoma and autoimmune diseases. Further investigations are needed to clarify the origin of these two benign lesions, including immunohistochemistry and embryological study.

The patient showed a gradual reduction in symptoms after surgery. This clinical improvement led to a progressive reduction in corticosteroid therapy until its discontinuation. At 1-year follow-up, the patient was free of RA symptoms. Currently, three years after surgery, the patient shows no joint symptoms, and no synovitis was reported at ultrasound. The continuation of follow-up is necessary to intercept any RA recurrence.

## 3. Discussion

The thymus gland plays a crucial role in T-cell differentiation and maturation and in the selection of non-self-reactive clones. The thymus reaches its maximum development at puberty and progressively undergoes fatty involution. Meunier et al. [1] reported that incomplete thymic involution was more frequent in patients with SS and RA rather than in the control group. Regarding RA patients, they observed that incomplete thymic involution (intended as residual thymus tissue measuring more than 7 mm on the short axis in the axial slice) was associated with the administration of biotherapy; this finding is interpreted as a greater severity of disease. Incomplete involution could be the substratum for MTC development.

The MTC is an uncommon acquired cyst originating from incomplete thymic involution that often presents follicular lymphoid hyperplasia. This finding probably originates from the transformation of medullary duct epithelium-derived structures as proposed by Suster and Rosai [4].

The MTCs are entities associated with autoimmune diseases like RA and SS. In addition to rheumatologic diseases, there are a whole range of autoimmune or immune-mediated diseases [11,12] associated with MTC, as well as neoplastic diseases [13], of the thymus or other immune-altering conditions.

Izumi and colleagues [2] presented a case series of four resected MTCs. Two were associated with Sjögren syndrome and the other two were associated with recurrent fever without any diagnosed autoimmune disease. All patients underwent extended thymectomy and histological analysis and showed the same findings described by Suster and Rosai, including lymphoid tissue organized in germinal centers; in addition, some HC showed cystic degeneration and appeared to be connected to other cystic spaces. No information was provided regarding disease activity and symptoms changing after surgery.

In the case of neoplastic disease association, MTC development is explained by inflammation of the microenvironment rather than the tumor itself [4]. In the case of myasthenia gravis (MG), as addressed by Gill et al. [12], the authors deal with the report of MG related to MTC (without other thymic lesions) and report other five cases published in the literature; this element has a particular relevance given that traditionally the thymic alterations associated with myasthenia gravis were thymoma, thymic hyperplasia, and thymic involution [14]. These findings contribute to stimulate further research on the link between MG and thymus.

In the international literature, there is only one other case of surgically treated MTC with high SUV at PET-CT with F^18^ FDG in a patient with RA symptoms [15], and in an additional report without mention of the F^18^ FDG investigation [16].

The paper by Matsumoto et al. [15] described a case of resected MTC with high FDG uptake, in which RA symptoms improved. Their interpretation is the arrest of the progression of symptoms after surgical resection. Unfortunately, only the abstract of the paper is available in English language and further details about the case are not readily reachable.

Kawamoto et al. [16] described a patient who underwent surgical resection of MTC, with a preoperative positivity for anti-CCP antibodies, normal rheumatoid factors, and no joint manifestations; this patient was therefore diagnosed with preclinical RA. Data regarding the follow-up of the rheumatological disorder are not available.

Other two papers report MTC with high SUV at PET-CT with F^18^ FDG, of which one is accompanied by the diagnosis of Sjögren syndrome, with a histological finding of lymphoid hyperplasia [17], and the other is characterized by epithelioid granulation [18].

The development of MTC from the medullary duct epithelium is stimulated by an inflammatory process; however, there are reports where MTC excision improves symptoms of autoimmune diseases [15,19], so the thymus itself could have a role in the pathogenesis of these diseases.

The meaning of F^18^ FDG uptake is still unclear, but this metabolic activity could be related to germinal cell proliferation, as found in lymphoid organs after antigen presentation. After immunohistochemical studies, Nakamura et al. [13] formulated the hypothesis that a maturation gradient of the HC exists from the cortical area toward the medullary zone, where they assume that the characteristic swirling forms. According to this assumption, MTC represents the cystic dilation of the inner layer of the HC in which an inflammatory cause can provoke epithelial proliferation and cellular crowding, as found by Izumi et al. [2].

Considering the available literature, knowledge about the pathogenesis of RA, and the role of the thymus in immune tolerance, we assume that there is a disruption of immune tolerance in the thymus and that self-reactive T-lymphocyte clones proliferate in the germinal centers that form the cyst. Some of the involved antigens could be those identified by Berthelot. After cyst removal, the production of self-reactive T lymphocytes decreases, leading to clinical improvement. At present, this sequence of events is only a conjecture and further studies are needed.

Recently, a retrospective observational study [20] of 1146 patients compared patients who underwent thymectomy with patients who underwent cardiothoracic surgery without thymectomy (control). The risk of cancer development was two-fold greater in patients who underwent thymectomy at 5 years, with a lower prevalence at 20 years. Moreover, “thymectomy appeared to have transiently and modestly increased the risk of autoimmune disease”. This result, even if derived from a retrospective study, suggests a lifelong role of the thymus in immunosurveillance and a more complex position than expected in autoimmune disease pathogenesis. These recent results allow musings about the type of surgery to be performed for this pathology. In the report provided by Matsumoto, an extended thymectomy was performed. Kondo et al. [19] warn about the possibility of having an association between MTC and thymoma or thymic carcinoma, and the risk of recurrence in case of incomplete cyst removal; accordingly, total thymectomy seems to be the best therapeutical choice. We believe that in the era of extensive computed tomography use, it is unlikely that a separate mass in the anterior mediastinum is not identified. Considering the aforementioned findings and the slight increase in the risk of autoimmune diseases, a patient-centered assessment is recommended before performing a radical thymectomy. Considering the benign nature of the disease and the increased morbidity of total thymectomy (e.g., phrenic nerve lesion), excision of the cyst could be a valid option.

The current literature suggests that the cause of inflammation in the case of MTC is always investigated; in the future, after further studies on this subject, there might be a proposal to rule out the presence of an anterior mediastinal mass in the case of clinically manifest autoimmune disease, namely, RA, SS, and Sjogren’s syndrome, or in a group of them with certain characteristics. A cause-and-effect relationship between MTC and autoimmune diseases is still a long way off, but given the symptomatic improvements after surgical removal, there may be a subset of patients in whom MTC plays a pathogenetic role and could benefit from surgical resection.

To test this hypothesis, it is necessary that a larger number of patients is investigated, ideally starting a prospective study to better assess clinical features before surgery, and possibly identifying a subset of patients with MTC, in whom RA has peculiar characteristics.

## 4. Conclusions

The MTC are uncommon acquired cysts that rarely show F^18^ FDG uptake. The association between MTC and RA is poorly explored. This report is limited to one patient’s experience; even if they find their match in the literature, these findings can not be generalized, so prospective studies are needed. In our opinion, this experience helps to shed light on the pathogenesis of acquired thymic cysts and their link with immune-mediated diseases, which encourages further research on this topic focused on the MTC molecular environment, a larger epidemiology to better correlate RA (and other autoimmune diseases) and MTC, and the significance of FDG uptake in these mediastinal masses.

## Data Availability

Data is contained within the article.
